# Rapid Identification of *Vicatia thibetica* de Boiss and Quantitative Analysis of the Content of Six Chemical Components Based on Near-Infrared Spectroscopy

**DOI:** 10.3390/molecules30091867

**Published:** 2025-04-22

**Authors:** Yunta Zhang, Jian Li, Jin Sun, Tian Xia, Yonglin Hai, Jian Li, Yongcheng Yang, Conglong Xia

**Affiliations:** 1 College of Pharmacy, Dali University, Dali 671000, China; 18487680093@163.com (Y.Z.); li178294@163.com (J.L.); 18523507753@163.com (J.S.); 15687203738@163.com (T.X.); hai520874@163.com (Y.H.); 2 State Key Laboratory of Bioreactor Engineering, Shanghai Key Laboratory of New Drug Design, East China University of Science and Technology, Shanghai 200237, China; jianli@ecust.edu.cn

**Keywords:** Apiaceae, NIR, HPLC-UV, origin identification, OPLS-DA, ANN

## Abstract

This study developed a rapid, non-destructive method combining near-infrared (NIR) spectroscopy with chemometric techniques (OPLS-DA, ANN, and PLS) to accurately identify the geographic origin and quantify six key chemical components of *V. thibetica* rhizomes. The results demonstrated that the combination of NIR spectroscopy, OPLS-DA, and ANN successfully and accurately distinguished *V. thibetica* from three distinct origins. Additionally, combining partial least squares (PLS) and NIR spectroscopy, the contents of chlorogenic acid, isochlorogenic acid A, isochlorogenic acid C, umbelliferone (7-hydroxycoumarin), senkyunolide I, and ligustilide measured by HPLC-UV were used as reference values to predict the contents of the six chemical components in *V. thibetica*, and spectral preprocessing methods optimized the model. The correlation coefficients of the final quantitative model for the contents of the six components in *V. thibetica* were between 0.7852 and 0.9538, the root mean square error of calibration (RMSEC) was between 0.0027 and 0.2530, and the root mean square error of prediction (RMSEP) was between 0.0031 and 0.4240. The results suggest that NIR spectroscopy combined with OPLS-DA and ANN can be used as a rapid and accurate method to evaluate the quality of *V. thibetica* herbs.

## 1. Introduction

*Vicatia thibetica* de Boiss (“Xizangaoruqin” in Chinese) is an essential medicinal and food plant of the genus *Vicatia* in the family Umbelliferae. Its root, commonly referred to as Xigui, serves as both the medicinal and edible portion. This species is renowned for expelling wind, eliminating dampness, dispersing cold, and relieving pain, primarily distributed in China’s Yunnan, Sichuan, and Tibet [1,2]. Recent studies have revealed its pharmacological effects including anti-inflammatory, anti-aging, anti-fatigue, antioxidant, anti-dysmenorrhea, and immunity-enhancing properties [3]. Previous research [3,4,5,6,7] has demonstrated that various secondary metabolites—particularly flavonoids and organic acids, like chlorogenic acid, isochlorogenic acid A, isochlorogenic acid C, umbelliferone, senkyunolide I, and ligustilide—are closely associated with these therapeutic benefits. Consequently, quantifying these components is crucial for quality evaluation. However, currently, the quality control of the plant mainly focuses on only one or a few components. For example, some studies only use ferulic acid [8] as the key quality control index, which obviously ignores the pharmacological action system jointly established by many other important components. This plant is mainly distributed in Yunnan, Sichuan, and Tibet regions. The geographical environments in different producing areas vary significantly, including soil types, climatic conditions (temperature, precipitation, light, etc.), altitude, and so on. These differences in environmental factors are highly likely to have an impact on the content and types of chemical components in the plant. Academician Huang Luqi believes that the quality of medicinal materials is affected by different producing areas, resulting in a decrease in the similarity of fingerprint components and significant differences in the content of effective components [9]. Therefore, the influence of the producing area factor on the quality of the plant is of great importance and cannot be ignored. Traditional quality control methods for *V. thibetica* include HPLC [7,8,10] and ultra-high performance liquid chromatography-quadrupole time-of-flight mass spectrometry (UPLC-Q-TOF-MS) technology [11], Still, the complex sample preparation process and extended analysis time of these traditional methods make it difficult to achieve rapid routine analysis of large quantities of *V. thibetica* samples and real-time monitoring.

Compared to traditional methods, near-infrared (NIR) spectroscopy offers significant advantages, such as fast analysis speed, high accuracy, simple operation, no need for additional reagents, and environmental friendliness [12]. NIR combined with chemometrics has been successfully applied to the identification and content prediction of Chinese herbal medicines; for example, Zhao et al. developed a rapid NIRS-chemometrics method combining OPLS-DA/RF (100% species/94.4% origin identification) and PLS modeling (R = 0.90–0.99 for triterpenoids) to replace traditional subjective and chromatographic methods for *Alismatis Rhizoma* quality control [13], Lauß et al. developed a non-destructive NIRS-PLS method for rapid quantification of anti-inflammatory boswellic acids (AKBA/KBA) in *Boswellia* resins across seven species, achieving satisfactory prediction (RMSEP = 0.74%, R^2^ = 0.79 for AKBA) as a green alternative to HPLC analysis [14], and Liu et al. developed a rapid NIRS-based method combining chemometrics (HCA/PLS-DA/ANN/SVM/ELM for identification; PCR/SVR/PLSR/ANN/ELM for quantification) to authenticate *Panax notoginseng* and detect three common adulterants (Rhizoma Curcumae, Curcuma longa, Rhizoma Atractylodis Macrocephalae) [15]. NIR spectroscopy (12,000–4000 cm^−1^) captures overtones and combination bands of fundamental vibrations from CH, NH, OH, and SH functional groups [16,17]. However, NIR spectra exhibit broad peaks, weak absorption, and low spectral selectivity, necessitating multivariate analysis for quantitative applications. Common chemometric approaches include partial least squares (PLS), artificial neural networks (ANN), multiple linear regression, principal component analysis, ridge regression, and support vector machines. Among these, PLS remains the predominant linear regression method, while ANN is widely adopted for nonlinear modeling [16]. Furthermore, orthogonal partial least squares-discriminant analysis (OPLS-DA) has emerged as a powerful supervised pattern recognition technique for quality assessment and origin tracing due to its superior handling of intraclass variation. OPLS-DA uniquely manages both correlated and uncorrelated spectral variations while identifying biologically relevant discriminative markers [18,19]. Collectively, NIR spectroscopy coupled with these chemometric strategies represents a critical technological solution for achieving rapid, nondestructive, and environmentally friendly quality evaluation of *V. thibetica*.

This study aimed to develop a rapid, accurate, objective, and cost-effective quality assessment method for *V. thibetica*. To achieve this objective, we investigated the combination of NIR spectroscopy with OPLS-DA and ANN to accurately discriminate *V. thibetica* samples from three geographical origins. Subsequently, we employed PLS regression coupled with NIR spectroscopy to predict the concentrations of six bioactive compounds in *V. thibetica*, using HPLC-measured values of chlorogenic acid, isochlorogenic acid A, isochlorogenic acid C, umbelliferone, senkyunolide I, and ligustilide as reference standards. Various spectral preprocessing techniques were systematically evaluated to optimize model performance through comprehensive spectral pretreatment strategies.

## 2. Results and Discussions

### 2.1. V. thibetica Identification Based on NIR Spectroscopy and Chemometrics

#### 2.1.1. NIR Spectral Properties of *V. thibetica*

**Figure 1:**
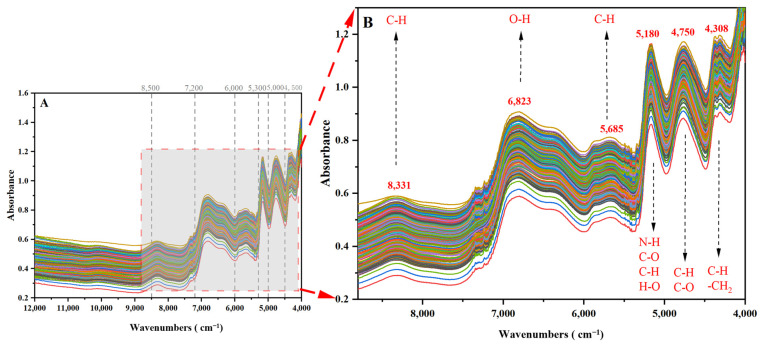
Original plots of 88 batches of *V. thibetica* samples (3 replicates): (**A**) Full-range absorbance spectra (12,000–4000 cm^−1^) displaying characteristic vibrational bands; (**B**) Characteristic spectral regions (8800–4200 cm^−1^) with annotated functional group assignments: 8331 cm^−1^: C-H (terpenoids); 6823 cm^−1^: O-H (phenolic acids); 5685 cm^−1^: C-H (terpenoids); 5180 cm^−1^: N-H/C=O (proteins/vitamin C); 4750 cm^−1^: C-H (Flavonoid); 4308 cm^−1^: -CH_2_ (polysaccharide).

As shown in Figure 1, *V. thibetica* exhibits characteristic absorption bands within the 12,000–4000 cm⁻^1^ range, with prominent peaks at 8331 cm⁻^1^, 6823 cm⁻^1^, 5685 cm⁻^1^, 5180 cm⁻^1^, 4750 cm⁻^1^, and 4308 cm⁻^1^. These spectral features primarily arise from fundamental, overtone, and combination vibrations of C-H, N-H, and O-H functional groups [20]. While the 88 sample batches displayed similar spectral profiles, origin differentiation proved challenging using raw spectra due to overlapping signals caused by instrumental noise and light scattering effects [21]. Notably, there were six distinct absorption peaks in the range of 8800–4200 cm^−1^: the weak absorption peaks observed near 8800–8000 cm^−1^ may be caused by the diploid absorption of the stretching vibration of the C-H bond [22]. The absorption peaks in the range of 7200~6500 cm^−1^ are related to the diploid stretching vibrations of the O-H bond, which may be associated with the presence of sugars and flavonoids [23]. The absorption peaks at 6000~5300 cm^−1^ can be attributed to the C-H bonds of flavonoids [24]. Significant absorption peaks in the 5300~5000 cm^−1^ region are associated with multiplicative absorption of N-H, C-H, O-H, and C=O bonds, mainly present in compounds, such as vitamin C, flavonoids, sugars, and proteins [25]. The absorption peaks at 5000~4500 cm^−1^ are related to the stretching vibration of C-H and C=O bonds and may be the absorption peaks of flavonoids [26]. The absorption peaks at 4400~4200 cm^−1^ are related to the stretching vibration of the C-H bond and the deformation vibration of -CH_2_, which may be the absorption peaks of polysaccharides and saponins [27]. The weaker absorption peaks at 6400~6100 cm^−1^ may be related to the two-fold absorption of hydrogen bonds [28]. These analytical results provide a necessary theoretical basis for further understanding the chemical composition and properties of *V. thibetica*.

#### 2.1.2. Identification of *V. thibetica* by OPLS-DA

**Figure 2:**
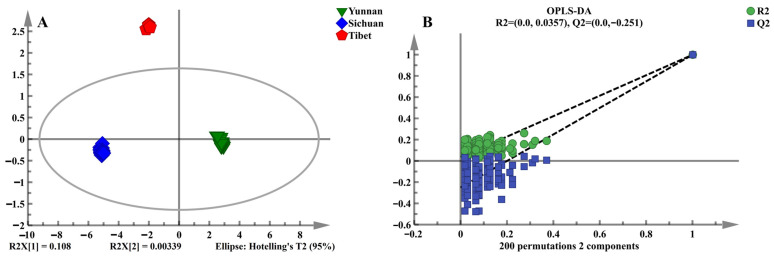
Multivariate statistical analysis of *V. thibetica* samples from different origins: (**A**) OPLS-DA score plots of *V. thibetica* from three origins; (**B**) 200 validation plots of three origins.

OPLS-DA was utilized to identify *V. thibetica* from different origins. Compared with other supervised chemometric methods, OPLS-DA can account for uncorrelated (orthogonal) variability in the spectra [29]. In this study, the model parameters were R^2^X = 0.998, R^2^Y = 0.996, and Q^2^ = 0.994, indicating that the model fit is stable and reliable. Figure 2A shows the score plot of the OPLS-DA model, clearly demonstrating that *V. thibetica* from different origins clustered into distinct categories. This indicates that the model has excellent discriminatory ability for the three origins of *V. thibetica*.

To validate the model, permutation testing was performed with 200 iterations. The results (Figure 2B) showed that the permuted R^2^ (0.0, 0.0357) and Q^2^ (0.0, −0.251) values were significantly lower than the original values (right side of the plot). This confirms that the OPLS-DA model exhibits no overfitting and has strong predictive power for classifying *V. thibetica* from the three sources.

#### 2.1.3. ANN Modeling to Identify *V. thibetica* from Different Origins

**Figure 3:**
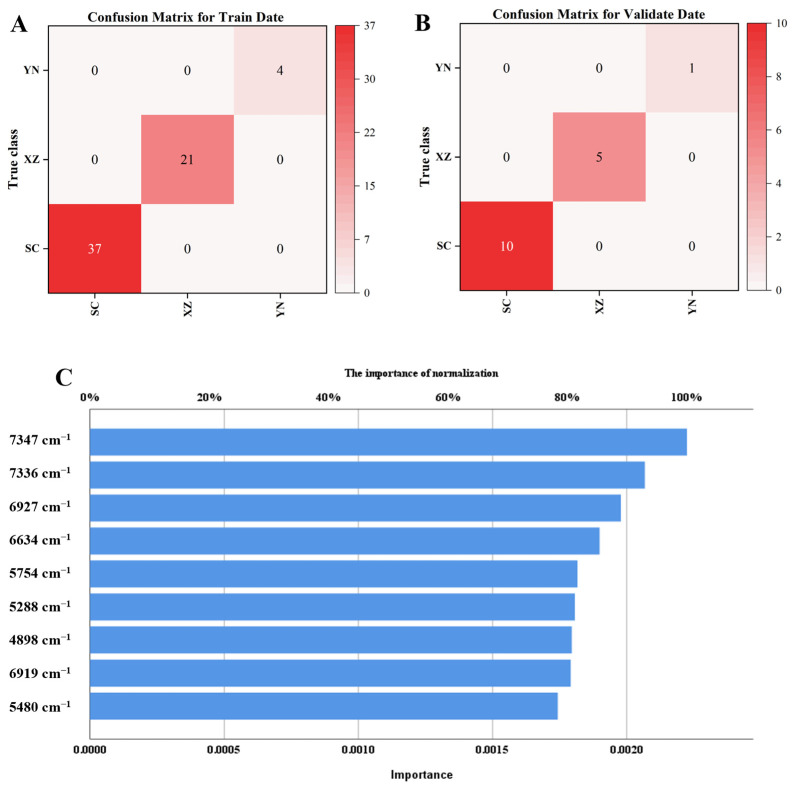
(**A**) Confusion matrix plot for the training set; (**B**) Confusion matrix plot for the validating set; (**C**) ANN variable importance analysis.

ANN is a highly efficacious tool in spectral data analysis that consists of multiple neuron layers capable of handling complex nonlinear problems with computational, nonlinear fitting, self-learning, and fault-tolerant properties [30]. Cui et al. successfully established an origin traceability method for *Crataegus pinnatifida* var. *major* in six provinces using near-infrared combined with ANN modelling [31]. However, although ANN can perform effective model-based learning when dealing with large-scale datasets, it is prone to overfitting when the amount of data is limited [32]. In this study, NIR data in the range of 8800–4200 cm^−1^ were used for MLP-ANN analysis. The 88 samples were partitioned into training, validation, and test sets in a ratio of 7:2:1, specifically comprising 70.5% (62 samples) as the training set, 18.2% (16 samples) as the validation set, and the remaining 11.4% (10 samples) as the external test set. The network architecture consisted of 1194 neuron units in the input layer, 17 units in the hidden layer, and 3 units in the output layer. The results are shown in Table 1, with 100% classification accuracy in both the training and validation sets across all three sources. In the external test set, samples from the XZ origin were excluded due to insufficient quantity, while the remaining two origins (YN and SC) achieved perfect classification accuracy (100%). The confusion matrices for the training and validation sets are presented in Figure 3A,B, respectively. Although these results highlight the model’s strong initial discriminative performance, future studies should expand the sample size to enhance robustness, implement rigorous cross-validation techniques (e.g., k-fold validation), and validate using completely independent external datasets to improve the model’s generalizability and reliability.

Based on the variable importance analysis of near-infrared spectroscopy (Figure 3C), nine characteristic wavelengths were identified in descending order of significance: 7347 cm^−1^, 7336 cm^−1^, 6927 cm^−1^, 6634 cm^−1^, 5754 cm^−1^, 5288 cm^−1^, 4898 cm^−1^, 6919 cm^−1^, and 5480 cm^−1^. These wavelengths correspond to distinct spectral regions and bioactive constituents: (1) 4898 cm^−1^ and 5288 cm^−1^ (5300–4500 cm^−1^ range) are primarily associated with vitamin C and flavonoid compounds; (2) 5754 cm^−1^ and 5480 cm^−1^ (6000–5300 cm^−1^ range) exhibit characteristic absorption of flavonoids; (3) 6927 cm^−1^, 6634 cm^−1^, and 6919 cm^−1^ (7200–6000 cm^−1^ range) correlate with sugars, organic acids, and flavonoid derivatives; (4) 7347 cm^−1^ and 7336 cm^−1^ (8800–7200 cm^−1^ range) are indicative of polyphenolic compounds. Numerous studies [5,6,7,8,11] have confirmed that *V. thibetica* is rich in polysaccharides, organic acids, and flavonoids, which constitute the material basis of its efficacy. The characteristic bands identified in this study correspond well with these components, suggesting that polysaccharides, organic acids, and flavonoids play a crucial role in the origin authentication of *V. thibetica*.

### 2.2. Simultaneous Determination of Six Compounds in V. thibetica by HPLC-UV

This study validated the method’s stability, precision, reproducibility, recovery, and standard curves for the six components. As shown in Table 2, the relative standard deviation (RSD) values for precision, stability, and reproducibility of all six components were below 3%, demonstrating good linearity for each component with recoveries ranging from 90.71% to 111.31%. These results confirm that the method meets all requirements for quality analysis.

**Figure 4:**
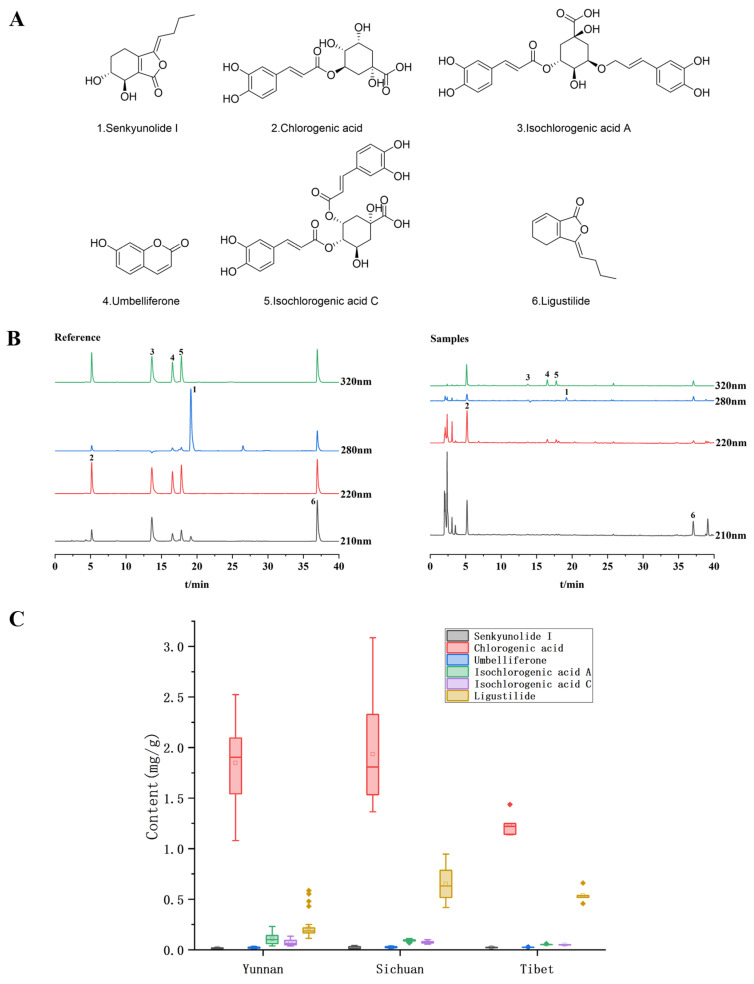
(**A**) Structures of six compounds in *V. thibetica*; (**B**) HPLC-UV chromatograms of reference substance and *V. thibetica* samples; (**C**) Contents of six chemical components in *V. thibetica*.

Figure 4A shows the chemical structures of the six components determined, and Figure 4B shows the chromatograms of the reference and samples determined at different wavelengths by HPLC-UV (1. senkyunolide I; 2. chlorogenic acid; 3. umbelliferone; 4. isochlorogenic acid A; 5. isochlorogenic acid C; 6. ligustilide). As can be seen from Figure 4C, the contents of the six compounds in the *V. thibetica* samples from different origins differed to some extent, with the chlorogenic acid contents of the samples from Yunnan and Sichuan being higher than those from Tibet and the ligustilide in the samples from Sichuan being higher than those from Yunnan and Tibet.

### 2.3. Quantitative Modeling of Six Chemical Components in V. thibetica Based on NIR Spectra

#### 2.3.1. Selection of Spectral Pre-Processing Methods

The spectral data were preprocessed using multiplicative signal correction (MSC), first derivative (FD), second derivative (SD), Savitzky-Golay filtering (SGF), and Norris derivative filtering (NDF). The performance of the partial least squares (PLS) models was evaluated based on three key metrics: the root mean square error of prediction (RMSEP), the root mean square error of calibration (RMSEC), and the coefficient of determination (R^2^). Model accuracy was considered higher when R^2^ values approached 1, while RMSEC and RMSEP values approached 0.

#### 2.3.2. Build and Validate NIR Calibration Models

This study systematically optimized NIR spectral preprocessing methods and successfully established quantitative analytical models for six active components in *V. thibetica*. As shown in Table 3, a combined preprocessing strategy comprising MSC, FD, and NDF enabled high-precision modeling of senkyunolide I (R^2^ = 0.8858), chlorogenic acid (R^2^ = 0.8035), umbelliferone (R^2^ = 0.9065), and ligustilide (R^2^ = 0.9538) within the spectral range of 7393.74–4315.91 cm^−1^. The RMSEC and RMSEP for these components remained below 0.0719 and 0.1070, respectively, validating the method’s reliability. Notably, the initial models for isochlorogenic acid A (R^2^ = 0.7988) and isochlorogenic acid C (R^2^ = 0.7852) exhibited suboptimal predictive performance. By replacing NDF with the SGF and refining four characteristic spectral sub-bands (4504.90–4342.91 cm^−1^, 4767.17–4539.61 cm^−1^, 6101.67–4774.88 cm^−1^, and 7189.32–6846.06 cm^−1^), the predictive accuracy of isochlorogenic acids was significantly enhanced. The optimized models achieved R^2^ values of 0.9450 (RMSEC = 0.0138, RMSEP = 0.0272) for isochlorogenic acid A and 0.9444 (RMSEC = 0.0085, RMSEP = 0.0015) for isochlorogenic acid C, demonstrating marked improvements.

This work represents the first successful co-optimization of NIR quantitative models for multi-component analysis in *V. thibetica*, offering a novel methodology for quality control of traditional Chinese medicinal materials. However, further optimization of preprocessing approaches and modeling parameters for isochlorogenic acids A and C remains essential, which will be prioritized in future research to advance analytical robustness and industrial applicability.

**Figure 5:**
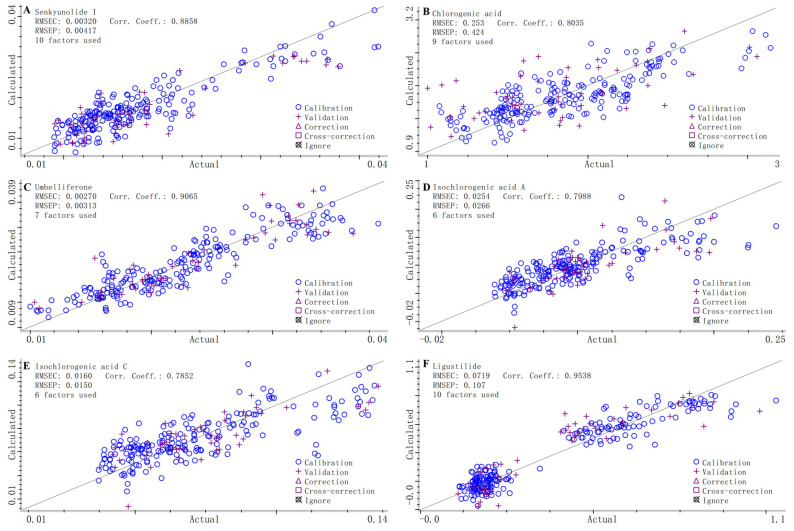
Quantitative calibration model for six components in *V. thibetica*: (**A**) senkyunolide I; (**B**) chlorogenic acid; (**C**) umbelliferone; (**D**) isochlorogenic acid A; (**E**) isochlorogenic acid C; (**F**) ligustilide.

Figure 5 shows the relationship between NIR and the content values determined by HPLC-UV for different compounds (e.g., senkyunolide I, chlorogenic acid, etc.) in *V. thibetica* samples. From the figure, it can be seen that for each compound, there was a significant linear relationship between the actual measured values and the calculated values of NIR spectra. In terms of the model evaluation index, the RMSEC of each compound was relatively small, indicating that the calibration model fit the calibration data well and accurately captured the data features. The RMSEP varied but was generally within an acceptable range, indicating that the model had a certain prediction ability for new data. The correlation coefficient values were mostly close to 1, further confirming the strong linear correlation between the actual and calculated values and highlighting the accuracy of the model.

Overall, the model based on NIR spectroscopy performed well in predicting the compound contents of *V. thibetica*, providing a reliable way to determine the compound contents of this sample quickly and efficiently. However, the model can be further optimized, such as by exploring better factor combinations, to improve its generality and prediction accuracy for complex samples.

**Figure 6:**
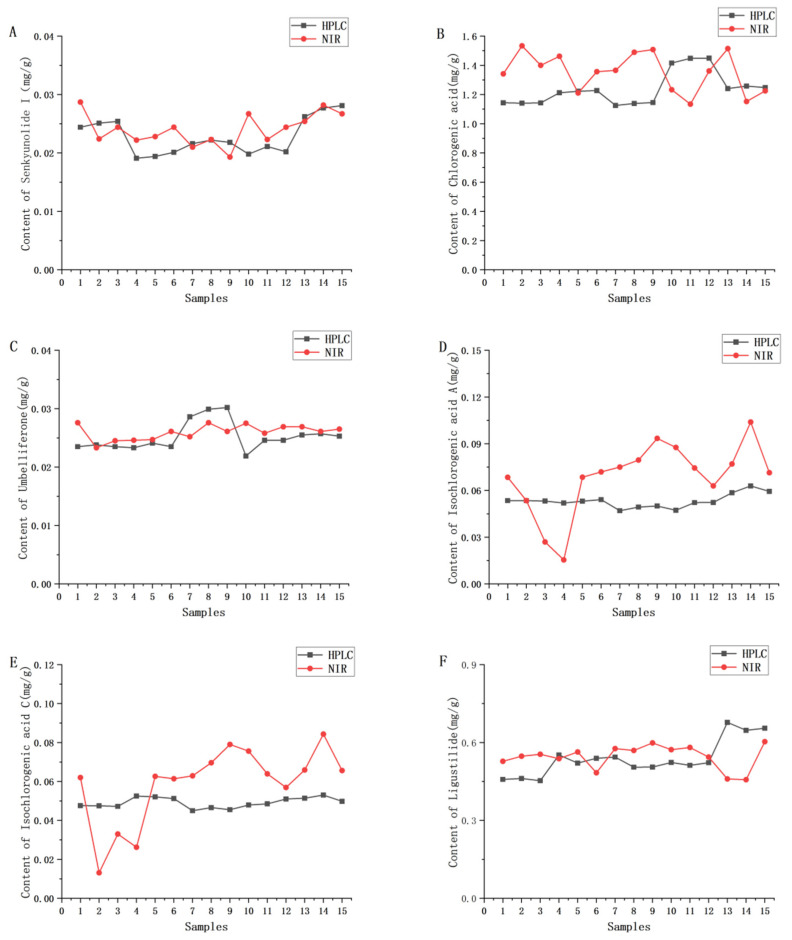
Comparison of HPLC-UV measured values and NIR predicted values of six chemical constituents in *V. thibetica* herbs: (**A**) senkyunolide I; (**B**) chlorogenic acid; (**C**) umbelliferone; (**D**) isochlorogenic acid A; (**E**) isochlorogenic acid C; (**F**) ligustilide.

Fifteen batches of samples not involved in the modelling were selected for external validation experiments to test the accuracy of the constructed model. Firstly, it can be observed from Figure 6 that the predicted values of three components, namely chlordecone I, umbelliferone, and ligustilide, were closer to the measured values among the components involved in the assay, and the trends of the two were basically the same. However, the predicted values of chlorogenic acid, isochlorogenic acid A, and isochlorogenic acid C showed some deviations from the measured values.

Further *t*-tests were performed on the predicted and real values of the components, and the test results were that the mean values of HPLC-measured values and NIR-predicted values of five components, namely senkyunolide I (*p* = 0.114), chlorogenic acid (*p* ≈ 0.058), umbelliferone (*p* ≈ 0.275), isochlorogenic acid C (*p* ≈ 0.094), and ligustilide (*p* = 0.728), were *p* > 0.05, with no significant difference, while the mean values of HPLC determination and NIR prediction of isochlorogenic acid A (*p* ≈ 0.023) were *p* < 0.05, with a significant difference. The above results indicated that NIR combined with PLS could effectively predict the contents of five components, namely senkyunolide I, chlorogenic acid, umbelliferone, isochlorogenic acid C, and ligustilide, in the samples of *V. thibetica*, while the quantitative model of isochlorogenic acid A failed to achieve the expected results.

## 3. Materials and Methods

### 3.1. Samples of V. thibetica

**Figure 7:**
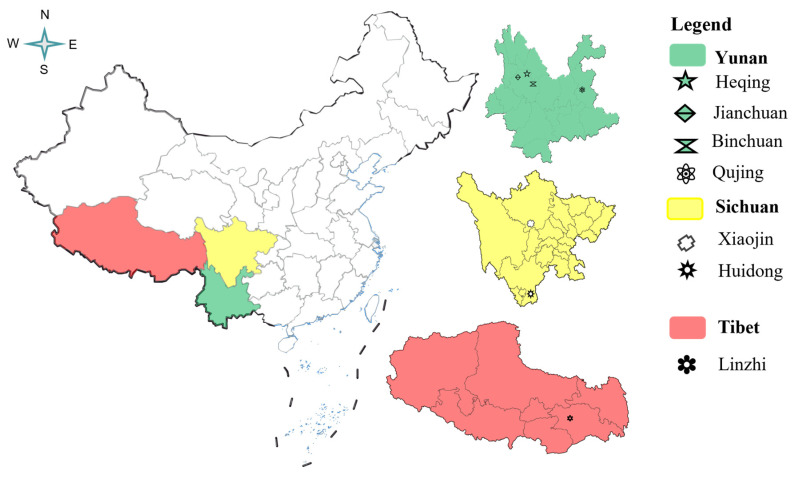
The geographical location of *V. thibetica* sample collection.

All 88 batches of samples were collected from three geographic regions in China (Yunnan, Sichuan, and Tibet) (Table 4; Figure 7). The samples were authenticated as *Vicatia thibetica* rhizomes by Prof. Xia Conglong (Dali University, Yunnan, China). After collection, the samples were rinsed with tap water and dried at room temperature under direct sunlight for 1 month (averaging 10 h of sunlight exposure per day). The dried material was then pulverized using a pulverizer (Model 800A, Hongtaiyang Electromechanical, Yongkang, China), sieved through an 80-mesh sieve, and stored in self-sealing bags. Finally, the powdered samples were labeled and kept in a desiccator for subsequent analysis.

### 3.2. Instruments and Reagents

The NIR spectrometer used in this study was purchased from Bruker (Berlin, Germany), and the HPLC system was obtained from Agilent Technologies (Santa Clara, CA, USA).

Reference standards (purity > 98%) were sourced as follows: chlorogenic acid (B20782) from Shanghai Yuanye Bio-Technology Co., Ltd. (Shanghai, China); isochlorogenic acid A (AZ21090101) from Chengdu Alfa Biotechnology Co., Ltd. (Chengdu, China); isochlorogenic acid C (I72610) from Shanghai Acmec Biochemistry Technology Co., Ltd. (Shanghai, China); umbelliferone (WKQ-00869), senkyunolide I (WKQ-0000636), and ligustilide (WKQ-0000246) from Sichuan Weikeqi Biological Technology Co., Ltd. (Chengdu, China). Chromatographic-grade reagents included methanol (Thermo Fisher Scientific, Waltham, MA, USA), acetonitrile (Shanghai Acmec Biochemical Technology Co., Ltd.), and phosphoric acid (Shanghai Macklin Biochemical Technology Co., Ltd., Shanghai, China). Ultrapure water was prepared using a purification system from Zhiang Instrument (Shanghai) Co., Ltd. (Shanghai, China)

### 3.3. NIR Spectral Acquisition

An appropriate amount of *V. thibetica* powder (approx. 3 g) was uniformly compacted in a transparent glass vial (maintaining consistent packing force for each measurement) and analyzed using a Bruker MATRIX-F NIR spectrometer (Lahr, Germany) operated in diffuse reflectance mode. Prior to sample measurement, the instrument was warmed up for 30 min and a background spectrum was acquired. Each sample was scanned 32 times with a spectral resolution of 8 cm^−1^ across the range of 12,000~4000 cm^−1^. To ensure analytical reproducibility, three independent spectral measurements were performed for each sample batch, with the averaged spectrum used for subsequent quantitative analysis.

### 3.4. Determination of Six Compounds by High Performance Liquid Chromatography

#### 3.4.1. Chromatographic Conditions

The HPLC-UV analysis was performed using an Agilent 1260 system under the following conditions: Separation was achieved on a Diamonsil C_18_ column (250 × 4.6 mm, 5 μm) with a gradient elution program consisting of acetonitrile (mobile phase B) and 0.1% aqueous phosphoric acid (mobile phase D). The gradient program was as follows: 0–10 min, 15–20% B; 10–20 min, 20–33% B; 20–35 min, 33–70% B; 35–40 min, 70–80% B; 40–48 min, 80–90% B. The injection volume was 10 μL with a flow rate of 1.0 mL/min. Detection wavelengths were set at 210 nm, 220 nm, 280 nm, and 320 nm, and the column temperature was maintained at 30 °C. Compound identification and quantification were performed by comparing retention times and UV absorbance profiles with reference standards.

#### 3.4.2. Sample Solution Preparation and Method Validation

Initially, 2.0 g of preprocessed *V. thibetana* powder (from Section 3.1) was homogenized with 30 mL of 70% methanol. The mixture was then subjected to ultrasonic extraction for 30 min using an ultrasonic cleaner (Model SB25-12D, Scientz Biotechnology Co., Ltd., (Ningbo, China) operating at 300 W. After cooling to room temperature, the weight loss was replenished with an appropriate volume of 70% methanol. The sample was centrifuged at 3000 rpm for 5 min using a benchtop high-speed centrifuge (Model H2-16K, Kecheng Instrument Equipment Co., Ltd., Changsha, China). The resulting solution was filtered through a 0.22-μm membrane filter and analyzed by HPLC-UV. Each sample was processed in triplicate, and the mean value was calculated as the actual content.

Standard compounds of *V. thibetica* (chlorogenic acid, isochlorogenic acid A, isochlorogenic acid C, umbelliferone, senkyunolide I, and ligustilide) were accurately weighed and dissolved in 70% methanol to prepare stock solutions with the following concentrations: chlorogenic acid (1.11 mg/mL), isochlorogenic acid A (0.06 mg/mL), isochlorogenic acid C (0.05 mg/mL), umbelliferone (0.02 mg/mL), senkyunolide I (0.03 mg/mL), and ligustilide (1.0 mg/mL). These solutions were then serially diluted with 70% methanol to appropriate concentrations for constructing calibration curves. Method validation was performed according to the 2020 edition of the Chinese Pharmacopoeia guidelines for analytical method validation of traditional Chinese medicine quality standards. The HPLC-UV method was evaluated for precision, reproducibility, and stability (analyzed at 0, 2, 4, 8, 12, and 24 h).

### 3.5. Chemometrics Analysis

#### 3.5.1. Preprocessing of NIR Spectra

Raw spectra obtained from NIR spectroscopic measurements contain not only useful sample information but also background interference and noise that may compromise model reliability and accuracy, making spectral preprocessing essential. In this study, we applied multiple preprocessing methods including MSC to address scattering effects caused by sample particle size and inhomogeneity, FD and SD treatments to eliminate baseline drift while enhancing resolution of overlapping peaks, along with SGF smoothing and NDF to improve signal-to-noise ratio. The SGF method serves as a versatile polynomial-based smoothing approach, whereas NDF is specifically designed for processing FD or SD transformed spectra in NIR analysis. Together, these techniques effectively minimize baseline drift and background interference while simultaneously improving spectral resolution and sensitivity [33,34].

#### 3.5.2. Discriminant Models

The OPLS-DA and ANN identification methods employed in this study represent supervised learning approaches that leverage extensive pre-labeled datasets for model training, enabling more accurate predictions compared to traditional non-hyperparameter pattern recognition methods like principal component analysis and cluster analysis. OPLS-DA enhances model accuracy and validity by systematically eliminating irrelevant data variations and removing category-independent noise interference. Model performance was rigorously evaluated using three key metrics: the coefficient of determination (R^2^X), the percentage of response variance explained by the model (R^2^Y), and the predictive power parameter (Q^2^), providing comprehensive assessment of both fitting quality and predictive capability [35].

ANNs represent information processing systems that simulate the human brain’s ability to process new data and acquire knowledge. These systems consist of interconnected simple processing units that collectively mimic the structure and functionality of biological neural networks. Characterized by self-organization, self-learning capabilities, robustness, fault tolerance, and nonlinear information processing, ANNs find widespread applications across diverse fields [36]. Among various architectures, the BP-ANN is particularly prominent as a multilayer feed-forward network comprising input, hidden, and output layers. This architecture employs forward signal propagation and backward error propagation, exhibiting exceptional nonlinear modeling capabilities that make it particularly suitable for solving complex mapping problems [37,38].

#### 3.5.3. Quantitative Models

In this study, 249 out of 264 collected sample spectra were selected for PLS quantitative modeling, with 200 samples allocated to the calibration set and 49 to the validation set. The PLS method was employed to establish a quantitative model for six bioactive components in *V. thibetica*, effectively projecting predictor (X, NIR spectral data) and response (Y, component concentrations) variables into a new latent space. This approach addresses multicollinearity issues in the predictor matrix—particularly when dealing with high-dimensional spectral data—while eliminating irrelevant spectral variations to enhance model performance [39,40]. Model efficacy was rigorously evaluated using R^2^ for both calibration and validation sets, along with RMSEP, which collectively assess predictive accuracy and robustness.

## 4. Conclusions

This study established, for the first time, a reliable method for identifying *V. thibetica* geographical origins by integrating NIR spectroscopy with chemometrics. Both OPLS-DA and ANN models showed strong classification performance in differentiating the three geographical origins, suggesting their potential utility in origin authentication studies. Beyond qualitative identification, NIR spectroscopy enabled rapid quantification of herbal constituents.

Complementary HPLC-UV analysis revealed significant origin-dependent variations in the concentrations of six key chemical components in *V. thibetica*. Building on these findings, we developed the first NIR-based quantitative calibration model using PLS regression for these six components, which yielded robust predictive performance. Together, these results highlight the value of combining NIR spectroscopy with multivariate analysis: this approach not only expands existing knowledge of *V. thibetica*’s chemical profile but also offers a rapid, simple, and reliable solution for geographical authentication and quality control. Furthermore, the methodology serves as a valuable reference for quality analysis of other medicinal and food products.

## Figures and Tables

**Table 1:** Results of artificial neural network prediction.

Sample Set	Actual Origin	Predicted Origin			Accuracy (%)
		Yunnan	Sichuan	Tibet	
Training	Yunnan	37	0	0	100.0
	Sichuan	0	21	0	100.0
	Tibet	0	0	4	100.0
	Total (%)	59.7	33.9	6.5	100.0
Validating	Yunnan	10	0	0	100.0
	Sichuan	0	5	0	100.0
	Tibet	0	0	1	100.0
	Accuracy (%)	62.5	31.3	6.3	100.0
Testing	Yunnan	8	0	0	100.0
	Sichuan	0	2	0	100.0
	Tibet	0	0	0	0.0
	Total (%)	80.0	20.0	0.0	100.0

**Table 2:** Tests of linear relationships.

Component	Regression	Linear Range (μg/mL)	Precision (RSD) (%)	Repeatability (RSD) (%)	Stability (RSD) (%)	Recovery (%)
Senkyunolide I	Y = 75.213X + 3.4951 R^2^ = 0.9992	0.3~9.0	0.67	2.86	0.36	105.51
Chlorogenic acid	Y = 15.35X − 30.69 R^2^ = 0.9992	22.2~244.2	1.14	0.93	0.33	111.31
Umbelliferone	Y = 83.873X + 6.6367 R^2^ = 0.9994	0.4~4.0	2.18	0.99	1.39	98.93
Isochlorogenic acid A	Y = 27.02X − 35.422 R^2^ = 0.9992	2.4~18.0	0.65	1.38	1.51	97.19
Isochlorogenic acid C	Y = 33.148X − 37.143 R^2^ = 0.9992	2.0~15.0	0.88	1.27	1.53	90.71
Ligustilide	Y = 14.813X + 0.8867 R^2^ = 0.9999	2.0~120.0	0.95	1.82	0.31	102.71

**Table 3:** Performance metrics of PLS models for six bioactive compounds under various spectral preprocessing methods (concentration units: mg/g dry weight).

Pre-Treatment Methods	-	Senkyunolide I	Chlorogenic Acid	Umbelliferone	Isochlorogenic Acid A	Isochlorogenic Acid C	Ligustilide
MSC	R^2^	0.8351	0.5525	0.9017	0.7026	0.6994	0.9447
	RMSEC	0.0038	0.3540	0.0028	0.0300	0.0185	0.0787
	RMSEP	0.0041	0.4050	0.0033	0.0268	0.0163	0.1080
	Factors	10	3	9	3	3	10
	Wavelength	11,539.95–4034.35 cm^−1^					
MSC + FD	R^2^	0.7286	0.4563	0.8834	0.7891	0.8902	0.9460
	RMSEC	0.0047	0.3780	0.0030	0.0259	0.0118	0.0776
	RMSEP	0.0058	0.4300	0.0038	0.0274	0.0155	0.1450
	Factors	4	2	4	4	6	6
	Wavelength	4524.18–4346.76 cm^−1^; 4767.17–4535.75 cm^−1^; 6375.51–4771.03 cm^−1^; 7146.90–6834.49 cm^−1^					
MSC + SD	R^2^	0.3148	0.1848	0.2753	0.2037	0.3369	0.7941
	RMSEC	0.0066	0.4170	0.0067	0.0413	0.0243	0.1460
	RMSEP	0.0083	0.4820	0.0067	0.0389	0.0228	0.1620
	Factors	1	1	1	1	1	1
	Wavelength	11,748.22–4045.92 cm^−1^					
MSC + SGF	R^2^	0.8218	0.7925	0.8919	0.7023	0.6993	0.9409
	RMSEC	0.0039	0.2590	0.0029	0.0300	0.0185	0.0811
	RMSEP	0.0041	0.3760	0.0033	0.0266	0.0163	0.1090
	Factors	9	10	8	3	3	9
	Wavelength	11,547.66–4042.07 cm^−1^					
MSC + FD + NDF	R^2^	0.8858	0.8035	0.9065	0.7988	0.7852	0.9538
	RMSEC	0.0032	0.2530	0.0027	0.0254	0.0160	0.0719
	RMSEP	0.0042	0.4240	0.0031	0.0266	0.0150	0.1070
	Factors	10	9	7	6	6	10
	Wavelength	7393.74–4315.91 cm^−1^					
MSC + SD + NDF	R^2^	0.8494	0.7088	0.8885	0.7744	0.7755	0.9083
	RMSEC	0.0036	0.3000	0.0029	0.0267	0.0163	0.1000
	RMSEP	0.0044	0.4090	0.0033	0.0284	0.0165	0.1170
	Factors	8	8	7	3	8	5
	Wavelength	6159.52–4308.19 cm^−1^; 7335.89–6680.21 cm^−1^					
MSC + FD + SGF	R^2^	0.7100	0.4540	0.8810	0.9450	0.9444	0.9442
	RMSEC	0.0049	0.3780	0.0030	0.0138	0.0085	0.0789
	RMSEP	0.0059	0.4310	0.0038	0.0272	0.0015	0.1430
	Factors	4	2	4	10	10	6
	Wavelength	4504.90–4342.91 cm^−1^; 4767.17–4539.61 cm^−1^; 6101.67–4774.88 cm^−1^; 7189.32–6846.06 cm^−1^					
MSC + SD + SGF	R^2^	0.3014	0.5873	0.9371	0.6707	0.6875	0.8895
	RMSEC	0.0066	0.3440	0.0022	0.0313	0.0188	0.1090
	RMSEP	0.0083	0.4360	0.0050	0.0377	0.0197	0.1610
	Factors	10	9	7	6	6	10
	Wavelength	9534.34–4412.33 cm^−1^; 11,343.24–9538.20 cm^−1^; 11,794.50–11347.10 cm^−1^					

Abbreviations: MSC (Multiplicative Scatter Correction), FD (First Derivative), SD (Second Derivative), SGF (Savitzky–Golay Filtering), NDF (Normalization by Division Factor). RMSEC and RMSEP values represent the root mean square error of calibration and prediction, respectively, in mg/g units.

**Table 4:** Information on the *V. thibetica* samples.

Region (Province)	Location	Number
Yunnan	Machang Village, Heqing County, Dali Bai Autonomous Prefecture	10
	Jianchuan County, Dali Bai Autonomous Prefecture, Yunnan	10
	Binchuan County, Dali Bai Autonomous Prefecture, Yunnan	5
	Bole Village, ZhanYi District, Qujing City	30
Sichuan	Xiaojin County in Aba Autonomous Prefecture, Sichuan	10
	Huidong County, Liangshan Yi Autonomous Prefecture	8
	Tangtang Township, Huidong County, Liangshan Yi Autonomous Prefecture, China	5
	Gaji Township, Huidong County, Liangshan Yi Autonomous Prefecture, China	5
Tibet	Bomi County, Linzhi City, Tibet	5

## Data Availability

The original contributions presented in this study are included in the article. Further inquiries can be directed to the corresponding authors.
